# P-1910. Long COVID Resolution Status and Health Impacts

**DOI:** 10.1093/ofid/ofae631.2071

**Published:** 2025-01-29

**Authors:** Christopher Kricorian

**Affiliations:** For Good Measure, Simi Valley, California

## Abstract

**Background:**

Many Americans continue to experience Long COVID (LC), which can affect a broad range of physical and mental functions. Understanding symptom prevalence is important to developing public health response.

**Methods:**

An online survey was conducted by the US Census Bureau (N=68,544). Self-reported health metrics (mobility, seeing, hearing and concentration) were compared between those reporting unresolved LC (LC-U), resolved LC (LC-R), previous short COVID (SC), and those not contracting COVID (NC). Data were analyzed using χ^2^ and between group contrast analysis.

**Results:**

Among those surveyed, 42.2% experienced SC, 41.3% did not experience COVID-19 (NC), 9.7% experienced LC-R, and 6.8% had LC-U. At least some mobility impairment was reported by 28.7% of NC respondents and 27.6% of LC-R (ns). LC-U reported significantly greater mobility impairment (38.1%, p< .05) and SC reported less (15.8%, p< .05). LC-U were most likely to report difficulty seeing (46.8%), followed by LC-R (37.2%), NC (30.5%), and SC (23.3%) (all comparisons p< .05). NC (19.5%) and LC-R (19.6%) were about equally likely to report any hearing difficulty (ns). LC-U reported significantly greater difficulty hearing (27.0%, p< .05) and SC reported significantly less (15.0%, p< .05). LC-U were most likely to report any difficulty concentrating or remembering (55.1%), followed by LC-R (40.4%), NC (28.9%) and SC (22.2%, all comparisons p< .05).

**Conclusion:**

LC-U respondents reported the greatest difficulty across all four variables while SCreported the least. NC and LC-R reported similar hearing and mobility challenges, but LC-R performed worse than NC on seeing and concentration. It seems paradoxical that SC respondents reported fewer challenges than NC, but perhaps those who experienced SC were healthier to begin with and thus less vigilant about avoiding COVID. Despite claiming resolved LC, LC-R still reported worse seeing and concentration than both SC and NC (albeit better than LC-U). Additional research is needed to examine both the factors associated with contracting LC and the long-term effects that may remain even after people believe they have recovered from LC.

**Disclosures:**

All Authors: No reported disclosures

